# 6-(4-Chloro­phen­yl)-7-phenyl-2,3-dihydro-1*H*-pyrrolizine-5-carbaldehyde

**DOI:** 10.1107/S1600536811031369

**Published:** 2011-08-11

**Authors:** Peter R. W. E. F. Keck, Dieter Schollmeyer, Stefan Laufer

**Affiliations:** a Eberhard-Karls-University Tübingen, Auf der Morgenstelle 8, 72076 Tübingen, Germany; b University Mainz, Institut of Organic Chemistry, Duesbergweg 10-14, 55099 Mainz, Germany

## Abstract

The 4-chloro­phenyl residue in the title compound, C_20_H_16_ClNO, is oriented at a dihedral angle of 53.6 (3)° towards the phenyl ring and 42.0 (9)° towards the pyrrole ring of the pyrrolizine template. The phenyl ring is oriented at a dihedral angle of 45.4 (4)° towards the pyrrole ring.

## Related literature

For the biological activity of aryl­pyrrolizines as mPGES-1 inhibitors, see: Liedtke *et al.* (2009 ▶). For dual COX/LOX inhibitors, see: Laufer (2001*a* ▶,*b* ▶).
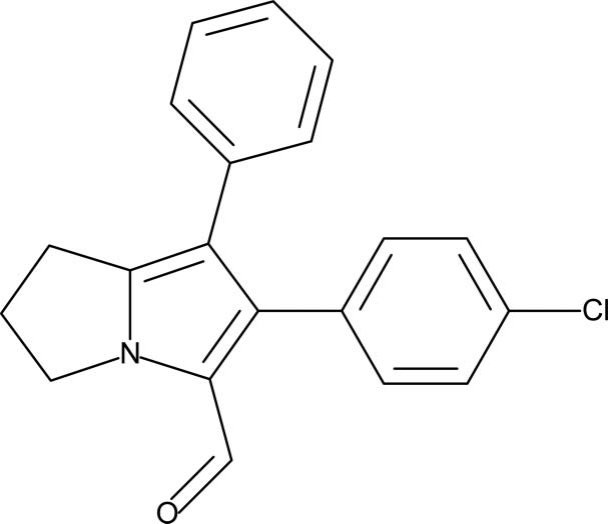


## Experimental

### 

#### Crystal data

C_20_H_16_ClNO
*M* *_r_* = 321.79
Monoclinic, 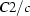

*a* = 21.1526 (13) Å
*b* = 11.5723 (9) Å
*c* = 17.1484 (12) Å
β = 130.843 (4)°
*V* = 3175.5 (4) Å^3^
*Z* = 8
Mo *K*α radiation
μ = 0.24 mm^−1^
*T* = 193 K
0.34 × 0.31 × 0.05 mm

#### Data collection

Stoe IPDS 2T diffractometer
9492 measured reflections
3804 independent reflections
2633 reflections with *I* > 2σ(*I*)
*R*_int_ = 0.036

#### Refinement

*R*[*F* ^2^ > 2σ(*F* ^2^)] = 0.040
*wR*(*F* ^2^) = 0.117
*S* = 1.02
3804 reflections
208 parameters
H-atom parameters constrained
Δρ_max_ = 0.18 e Å^−3^
Δρ_min_ = −0.38 e Å^−3^

### 

Data collection: *X-AREA* (Stoe & Cie, 2010 ▶); cell refinement: *X-AREA*; data reduction: *X-RED* (Stoe & Cie, 2010 ▶); program(s) used to solve structure: *SIR97* (Altomare *et al.*, 1999 ▶); program(s) used to refine structure: *SHELXL97* (Sheldrick, 2008 ▶); molecular graphics: *PLATON* (Spek, 2009 ▶); software used to prepare material for publication: *PLATON*.

## Supplementary Material

Crystal structure: contains datablock(s) I, global. DOI: 10.1107/S1600536811031369/bt5600sup1.cif

Structure factors: contains datablock(s) I. DOI: 10.1107/S1600536811031369/bt5600Isup2.hkl

Supplementary material file. DOI: 10.1107/S1600536811031369/bt5600Isup3.cml

Additional supplementary materials:  crystallographic information; 3D view; checkCIF report

## Figures and Tables

**Fig. 1.**
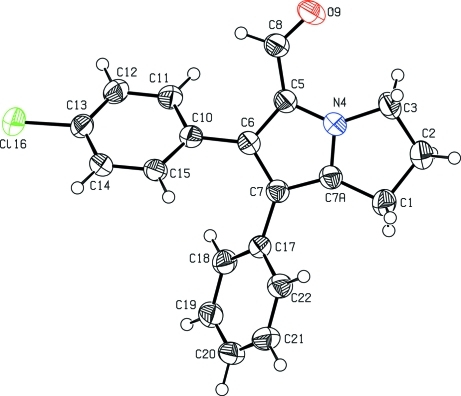
View of compound I. Displacement ellipsoids are drawn at the 50% probability level.

## supplementary crystallographic information

### Comment

Based on ML3000 (Laufer *et al.*, 2001*a*,*b*) as dual COX/LOX inhibitor, we synthesized and evaluated inhibitors for the microsomal prostaglandin E_2_ synthase-1 (mPGES-1) (Liedtke *et al.*, 2009). The title compound was synthesized to obtain a template with a reactive group in position 5 of the pyrrolizine moiety which lead to series of differend derivates of the arylpyrrolizine scaffold.

Towards the unsaturated and planar part of the pyrrolizine residue the 4-chlorophenyl residue is oriented at a dihedral angle of 42.0 (9)° and the plain phenyl ring is oriented at a dihedral angle of 45.4 (4)°. The two phenyl rings are oriented at a dihedral angle of 53.6 (3)° and both centromers show a distance of 5.07 (6) Å. The distance between the *para* C atoms of the rings (C13, C20) is 6.85 (0) Å.

### Experimental

The compound was prepared by Vilsmeyer reaction. Phosphoryl chloride (0.484 ml, 5.31 mmol) is added dropwise to ice-cooled solution of 1.18 ml dimethylformamide and 6-(4-chlorophenyl)-7-phenyl-2,3-dihydro-1*H*-pyrrolizine (1.5 g, 5.11 mmol); the temperature is kept under 293 K during the addition. Then the mixture is stirred for 1 h at room temperature. Finally the mixture is heated to 333 K for 1 h. The mixture was cooled to 273 K, quenched by water and adjusted to pH 6 with aqueous sodium hydroxide solution 10%.

The product was collected as precipitated solid by filtration, was dissolved in dichloromethane and washed with water three times and finally dried over anhydrous sodium sulfate. The product was concentrated under vacuum and precipitated out of diisopropylic ether to yield 1.13 g (69%). Crystals of the title compound were obtained by slow evaporation of ethanol at room temperature.

### Refinement

Hydrogen atoms attached to carbons were placed at calculated positions with C—H = 0.95 Å (aromatic) or 0.99–1.00 Å (*sp*^3^ C-atom). All H atoms were refined with isotropic displacement parameters (set at 1.2–1.5 times of the *U*_eq_ of the parent atom).

### Crystal data

**Table:**

C_20_H_16_ClNO	*F*(000) = 1344
*M**_r_* = 321.79	*D*_x_ = 1.346 Mg m^−^^3^
Monoclinic, *C*2/*c*	Mo *K*α radiation, λ = 0.71073 Å
Hall symbol: -C 2yc	Cell parameters from 6848 reflections
*a* = 21.1526 (13) Å	θ = 2.5–29.7°
*b* = 11.5723 (9) Å	µ = 0.24 mm^−^^1^
*c* = 17.1484 (12) Å	*T* = 193 K
β = 130.843 (4)°	Plate, colourless
*V* = 3175.5 (4) Å^3^	0.34 × 0.31 × 0.05 mm
*Z* = 8	

### Data collection

**Table:**

Stoe IPDS 2T diffractometer	2633 reflections with *I* > 2σ(*I*)
Radiation source: sealed X-ray tube, 12 x 0.4 mm long-fine focus	*R*_int_ = 0.036
graphite	θ_max_ = 28.0°, θ_min_ = 2.6°
Detector resolution: 6.67 pixels mm^-1^	*h* = −27→27
rotation method scans	*k* = −15→13
9492 measured reflections	*l* = −22→16
3804 independent reflections	

### Refinement

**Table:**

Refinement on *F*^2^	Primary atom site location: structure-invariant direct methods
Least-squares matrix: full	Secondary atom site location: difference Fourier map
*R*[*F*^2^ > 2σ(*F*^2^)] = 0.040	Hydrogen site location: inferred from neighbouring sites
*wR*(*F*^2^) = 0.117	H-atom parameters constrained
*S* = 1.02	*w* = 1/[σ^2^(*F*_o_^2^) + (0.0654*P*)^2^ + 0.4776*P*] where *P* = (*F*_o_^2^ + 2*F*_c_^2^)/3
3804 reflections	(Δ/σ)_max_ = 0.001
208 parameters	Δρ_max_ = 0.18 e Å^−^^3^
0 restraints	Δρ_min_ = −0.38 e Å^−^^3^

### Special details

**Table:**

Geometry. All e.s.d.'s (except the e.s.d. in the dihedral angle between two l.s. planes) are estimated using the full covariance matrix. The cell e.s.d.'s are taken into account individually in the estimation of e.s.d.'s in distances, angles and torsion angles; correlations between e.s.d.'s in cell parameters are only used when they are defined by crystal symmetry. An approximate (isotropic) treatment of cell e.s.d.'s is used for estimating e.s.d.'s involving l.s. planes.
Refinement. Refinement of *F*^2^ against ALL reflections. The weighted *R*-factor *wR* and goodness of fit *S* are based on *F*^2^, conventional *R*-factors *R* are based on *F*, with *F* set to zero for negative *F*^2^. The threshold expression of *F*^2^ > σ(*F*^2^) is used only for calculating *R*-factors(gt) *etc*. and is not relevant to the choice of reflections for refinement. *R*-factors based on *F*^2^ are statistically about twice as large as those based on *F*, and *R*- factors based on ALL data will be even larger.

### Fractional atomic coordinates and isotropic or equivalent isotropic displacement parameters (Å^2^)

**Table:**

	*x*	*y*	*z*	*U*_iso_*/*U*_eq_	
C1	0.62354 (11)	0.11928 (15)	0.31943 (15)	0.0441 (4)	
H1A	0.5719	0.0923	0.3037	0.053*	
H1B	0.6721	0.0780	0.3810	0.053*	
C2	0.61862 (13)	0.10249 (16)	0.22614 (16)	0.0510 (4)	
H2A	0.6461	0.0294	0.2326	0.061*	
H2B	0.5597	0.1007	0.1610	0.061*	
C3	0.66451 (11)	0.20673 (16)	0.22833 (15)	0.0451 (4)	
H3A	0.7241	0.1889	0.2663	0.054*	
H3B	0.6379	0.2328	0.1578	0.054*	
N4	0.65521 (8)	0.29317 (12)	0.28297 (10)	0.0375 (3)	
C5	0.66339 (10)	0.41119 (14)	0.29645 (12)	0.0369 (3)	
C6	0.64365 (9)	0.43919 (13)	0.35826 (12)	0.0351 (3)	
C7	0.62553 (9)	0.33471 (14)	0.38334 (12)	0.0349 (3)	
C7A	0.63337 (9)	0.24695 (14)	0.33439 (12)	0.0367 (3)	
C8	0.69420 (10)	0.48273 (16)	0.25951 (13)	0.0423 (4)	
H8	0.7003	0.5629	0.2748	0.051*	
O9	0.71332 (9)	0.44865 (13)	0.20975 (11)	0.0543 (3)	
C10	0.64265 (9)	0.55686 (14)	0.39052 (12)	0.0349 (3)	
C11	0.60820 (10)	0.64988 (15)	0.32190 (13)	0.0411 (4)	
H11	0.5853	0.6372	0.2530	0.049*	
C12	0.60672 (11)	0.75998 (15)	0.35203 (14)	0.0435 (4)	
H12	0.5839	0.8225	0.3047	0.052*	
C13	0.63874 (10)	0.77793 (14)	0.45162 (14)	0.0401 (4)	
C14	0.67412 (10)	0.68840 (14)	0.52186 (13)	0.0387 (3)	
H14	0.6972	0.7020	0.5907	0.046*	
C15	0.67564 (9)	0.57881 (14)	0.49104 (12)	0.0361 (3)	
H15	0.6996	0.5171	0.5393	0.043*	
Cl16	0.63185 (3)	0.91463 (4)	0.48838 (4)	0.05391 (15)	
C17	0.59919 (9)	0.31301 (13)	0.44330 (12)	0.0343 (3)	
C18	0.53753 (10)	0.37916 (15)	0.42983 (14)	0.0408 (4)	
H18	0.5134	0.4426	0.3834	0.049*	
C19	0.51117 (11)	0.35325 (16)	0.48348 (15)	0.0460 (4)	
H19	0.4690	0.3988	0.4734	0.055*	
C20	0.54598 (11)	0.26142 (17)	0.55167 (14)	0.0483 (4)	
H20	0.5276	0.2435	0.5882	0.058*	
C21	0.60775 (12)	0.19570 (17)	0.56642 (14)	0.0486 (4)	
H21	0.6321	0.1328	0.6135	0.058*	
C22	0.63404 (11)	0.22143 (15)	0.51282 (13)	0.0416 (4)	
H22	0.6765	0.1759	0.5236	0.050*	

### Atomic displacement parameters (Å^2^)

**Table:**

	*U*^11^	*U*^22^	*U*^33^	*U*^12^	*U*^13^	*U*^23^
C1	0.0489 (9)	0.0362 (9)	0.0538 (10)	0.0032 (7)	0.0364 (8)	−0.0003 (7)
C2	0.0619 (11)	0.0429 (10)	0.0578 (11)	−0.0025 (8)	0.0434 (10)	−0.0094 (8)
C3	0.0520 (9)	0.0457 (9)	0.0490 (9)	−0.0016 (7)	0.0379 (8)	−0.0100 (8)
N4	0.0421 (7)	0.0388 (7)	0.0386 (7)	0.0002 (5)	0.0294 (6)	−0.0017 (6)
C5	0.0390 (7)	0.0386 (8)	0.0354 (8)	−0.0016 (6)	0.0254 (7)	−0.0023 (6)
C6	0.0361 (7)	0.0365 (8)	0.0337 (7)	0.0008 (6)	0.0232 (6)	−0.0010 (6)
C7	0.0355 (7)	0.0354 (8)	0.0354 (7)	0.0023 (6)	0.0239 (6)	0.0016 (6)
C7A	0.0386 (7)	0.0361 (8)	0.0396 (8)	0.0031 (6)	0.0274 (7)	0.0012 (7)
C8	0.0471 (8)	0.0455 (9)	0.0397 (8)	−0.0059 (7)	0.0308 (8)	−0.0039 (7)
O9	0.0679 (8)	0.0623 (9)	0.0545 (8)	−0.0101 (7)	0.0496 (7)	−0.0086 (6)
C10	0.0359 (7)	0.0352 (8)	0.0369 (8)	−0.0004 (6)	0.0253 (7)	0.0003 (6)
C11	0.0477 (8)	0.0389 (9)	0.0371 (8)	−0.0003 (7)	0.0279 (7)	0.0021 (7)
C12	0.0500 (9)	0.0348 (8)	0.0458 (9)	0.0020 (7)	0.0313 (8)	0.0062 (7)
C13	0.0452 (8)	0.0330 (8)	0.0518 (9)	−0.0034 (6)	0.0360 (8)	−0.0027 (7)
C14	0.0449 (8)	0.0397 (8)	0.0414 (8)	−0.0012 (7)	0.0325 (7)	−0.0022 (7)
C15	0.0403 (8)	0.0372 (8)	0.0372 (8)	0.0031 (6)	0.0281 (7)	0.0032 (6)
Cl16	0.0722 (3)	0.0334 (2)	0.0704 (3)	−0.00204 (19)	0.0529 (3)	−0.0052 (2)
C17	0.0361 (7)	0.0326 (8)	0.0366 (8)	−0.0022 (6)	0.0248 (7)	−0.0015 (6)
C18	0.0424 (8)	0.0369 (8)	0.0482 (9)	0.0026 (6)	0.0319 (8)	0.0024 (7)
C19	0.0486 (9)	0.0449 (9)	0.0595 (10)	−0.0057 (7)	0.0420 (9)	−0.0083 (8)
C20	0.0591 (10)	0.0515 (10)	0.0512 (10)	−0.0127 (8)	0.0434 (9)	−0.0072 (8)
C21	0.0592 (10)	0.0454 (10)	0.0467 (9)	−0.0022 (8)	0.0370 (9)	0.0062 (8)
C22	0.0452 (8)	0.0403 (9)	0.0427 (8)	0.0030 (7)	0.0302 (7)	0.0035 (7)

### Geometric parameters (Å, °)

**Table:**

C1—C7A	1.491 (2)	C10—C11	1.397 (2)
C1—C2	1.546 (3)	C11—C12	1.383 (2)
C1—H1A	0.9900	C11—H11	0.9500
C1—H1B	0.9900	C12—C13	1.380 (3)
C2—C3	1.533 (3)	C12—H12	0.9500
C2—H2A	0.9900	C13—C14	1.380 (2)
C2—H2B	0.9900	C13—Cl16	1.7440 (17)
C3—N4	1.469 (2)	C14—C15	1.382 (2)
C3—H3A	0.9900	C14—H14	0.9500
C3—H3B	0.9900	C15—H15	0.9500
N4—C7A	1.345 (2)	C17—C22	1.392 (2)
N4—C5	1.377 (2)	C17—C18	1.395 (2)
C5—C6	1.408 (2)	C18—C19	1.385 (2)
C5—C8	1.432 (2)	C18—H18	0.9500
C6—C7	1.417 (2)	C19—C20	1.383 (3)
C6—C10	1.475 (2)	C19—H19	0.9500
C7—C7A	1.395 (2)	C20—C21	1.385 (3)
C7—C17	1.476 (2)	C20—H20	0.9500
C8—O9	1.224 (2)	C21—C22	1.383 (3)
C8—H8	0.9500	C21—H21	0.9500
C10—C15	1.396 (2)	C22—H22	0.9500
			
C7A—C1—C2	102.13 (15)	C15—C10—C11	117.63 (15)
C7A—C1—H1A	111.3	C15—C10—C6	120.84 (14)
C2—C1—H1A	111.3	C11—C10—C6	121.52 (15)
C7A—C1—H1B	111.3	C12—C11—C10	121.46 (16)
C2—C1—H1B	111.3	C12—C11—H11	119.3
H1A—C1—H1B	109.2	C10—C11—H11	119.3
C3—C2—C1	105.23 (14)	C13—C12—C11	119.22 (16)
C3—C2—H2A	110.7	C13—C12—H12	120.4
C1—C2—H2A	110.7	C11—C12—H12	120.4
C3—C2—H2B	110.7	C12—C13—C14	120.94 (16)
C1—C2—H2B	110.7	C12—C13—Cl16	119.62 (14)
H2A—C2—H2B	108.8	C14—C13—Cl16	119.42 (13)
N4—C3—C2	101.81 (13)	C13—C14—C15	119.33 (15)
N4—C3—H3A	111.4	C13—C14—H14	120.3
C2—C3—H3A	111.4	C15—C14—H14	120.3
N4—C3—H3B	111.4	C14—C15—C10	121.41 (15)
C2—C3—H3B	111.4	C14—C15—H15	119.3
H3A—C3—H3B	109.3	C10—C15—H15	119.3
C7A—N4—C5	110.10 (14)	C22—C17—C18	118.26 (15)
C7A—N4—C3	113.20 (14)	C22—C17—C7	119.75 (14)
C5—N4—C3	136.70 (15)	C18—C17—C7	121.94 (15)
N4—C5—C6	106.74 (14)	C19—C18—C17	120.67 (16)
N4—C5—C8	122.86 (15)	C19—C18—H18	119.7
C6—C5—C8	130.15 (16)	C17—C18—H18	119.7
C5—C6—C7	107.71 (14)	C20—C19—C18	120.32 (16)
C5—C6—C10	125.36 (15)	C20—C19—H19	119.8
C7—C6—C10	126.92 (14)	C18—C19—H19	119.8
C7A—C7—C6	106.05 (14)	C19—C20—C21	119.58 (16)
C7A—C7—C17	122.87 (14)	C19—C20—H20	120.2
C6—C7—C17	131.02 (14)	C21—C20—H20	120.2
N4—C7A—C7	109.39 (14)	C22—C21—C20	120.13 (17)
N4—C7A—C1	110.50 (14)	C22—C21—H21	119.9
C7—C7A—C1	140.10 (16)	C20—C21—H21	119.9
O9—C8—C5	125.12 (17)	C21—C22—C17	121.02 (16)
O9—C8—H8	117.4	C21—C22—H22	119.5
C5—C8—H8	117.4	C17—C22—H22	119.5
			
C7A—C1—C2—C3	25.15 (18)	C6—C5—C8—O9	176.32 (17)
C1—C2—C3—N4	−25.45 (18)	C5—C6—C10—C15	−137.95 (16)
C2—C3—N4—C7A	16.96 (19)	C7—C6—C10—C15	41.8 (2)
C2—C3—N4—C5	−163.46 (18)	C5—C6—C10—C11	42.7 (2)
C7A—N4—C5—C6	−1.26 (18)	C7—C6—C10—C11	−137.55 (17)
C3—N4—C5—C6	179.14 (17)	C15—C10—C11—C12	0.1 (2)
C7A—N4—C5—C8	173.57 (15)	C6—C10—C11—C12	179.48 (15)
C3—N4—C5—C8	−6.0 (3)	C10—C11—C12—C13	−1.1 (3)
N4—C5—C6—C7	1.41 (17)	C11—C12—C13—C14	1.8 (3)
C8—C5—C6—C7	−172.90 (16)	C11—C12—C13—Cl16	−176.36 (13)
N4—C5—C6—C10	−178.77 (14)	C12—C13—C14—C15	−1.5 (2)
C8—C5—C6—C10	6.9 (3)	Cl16—C13—C14—C15	176.68 (12)
C5—C6—C7—C7A	−1.06 (17)	C13—C14—C15—C10	0.4 (2)
C10—C6—C7—C7A	179.13 (14)	C11—C10—C15—C14	0.2 (2)
C5—C6—C7—C17	−178.20 (15)	C6—C10—C15—C14	−179.16 (14)
C10—C6—C7—C17	2.0 (3)	C7A—C7—C17—C22	45.3 (2)
C5—N4—C7A—C7	0.60 (18)	C6—C7—C17—C22	−137.98 (18)
C3—N4—C7A—C7	−179.70 (13)	C7A—C7—C17—C18	−132.22 (17)
C5—N4—C7A—C1	179.45 (13)	C6—C7—C17—C18	44.5 (2)
C3—N4—C7A—C1	−0.85 (19)	C22—C17—C18—C19	−0.7 (2)
C6—C7—C7A—N4	0.30 (17)	C7—C17—C18—C19	176.81 (15)
C17—C7—C7A—N4	177.73 (14)	C17—C18—C19—C20	0.3 (3)
C6—C7—C7A—C1	−178.0 (2)	C18—C19—C20—C21	0.3 (3)
C17—C7—C7A—C1	−0.6 (3)	C19—C20—C21—C22	−0.4 (3)
C2—C1—C7A—N4	−15.54 (18)	C20—C21—C22—C17	−0.1 (3)
C2—C1—C7A—C7	162.8 (2)	C18—C17—C22—C21	0.7 (3)
N4—C5—C8—O9	2.8 (3)	C7—C17—C22—C21	−176.92 (16)

## References

[bb1] Altomare, A., Burla, M. C., Camalli, M., Cascarano, G. L., Giacovazzo, C., Guagliardi, A., Moliterni, A. G. G., Polidori, G. & Spagna, R. (1999). *J. Appl. Cryst.* **32**, 115–119.

[bb2] Laufer, S. (2001*a*). *Inflammopharmacology*, **9**, 101–112.

[bb3] Laufer, S. (2001*b*). *Inflammopharmacology*, **9**, 113–124.

[bb4] Liedtke, A. J., Keck, P. R. W. E. F., Lehmann, F., Koeberle, A., Werz, O. & Laufer, S. (2009). *J. Med. Chem.* **52**, 4968–4972. 10.1021/jm900481c 19719242

[bb5] Sheldrick, G. M. (2008). *Acta Cryst.* A**64**, 112–122. 10.1107/S0108767307043930 18156677

[bb6] Spek, A. L. (2009). *Acta Cryst.* D**65**, 148–155. 10.1107/S090744490804362X PMC2631630 19171970

[bb7] Stoe & Cie (2010). *X-AREA* and *X-RED* Stoe & Cie GmbH, Darmstadt, Germany.

